# IMPC-based screening revealed that *ROBO1* can regulate osteoporosis by inhibiting osteogenic differentiation

**DOI:** 10.3389/fcell.2024.1450215

**Published:** 2024-10-08

**Authors:** Xiangzheng Zhang, Yike Wang, Miao Zheng, Qi Wei, Ruizhi Zhang, Keyu Zhu, Qiaocheng Zhai, Youjia Xu

**Affiliations:** ^1^ The Osteoporosis Clinical Center, The Second Affiliated Hospital of Soochow University, Suzhou, China; ^2^ Department of Orthopaedics, The Second Affiliated Hospital of Soochow University, Suzhou, Jiangsu, China; ^3^ Division of Spine Surgery, The Quzhou Affiliated Hospital of Wenzhou Medical University, Quzhou People’s Hospital, Quzhou, China

**Keywords:** osteoporosis, IMPC, ROBO1, osteogenesis, inflammation

## Abstract

**Introduction:**

The utilization of denosumab in treating osteoporosis highlights promising prospects for osteoporosis intervention guided by gene targets. While omics-based research into osteoporosis pathogenesis yields a plethora of potential gene targets for clinical transformation, identifying effective gene targets has posed challenges.

**Methods:**

We first queried the omics data of osteoporosis clinical samples on PubMed, used International Mouse Phenotyping Consortium (IMPC) to screen differentially expressed genes, and conducted preliminary functional verification of candidate genes in human Saos2 cells through osteogenic differentiation and mineralization experiments. We then selected the candidate genes with the most significant effects on osteogenic differentiation and further verified the osteogenic differentiation and mineralization functions in mouse 3T3-E1 and bone marrow mesenchymal stem cells (BMSC). Finally, we used RNA-seq to explore the regulation of osteogenesis by the target gene.

**Results:**

We identified *PPP2R2A*, *RRBP1*, *HSPB6*, *SLC22A15*, *ADAMTS4*, *ATP8B1*, *CTNNB1*, *ROBO1*, and *EFR3B*, which may contribute to osteoporosis. *ROBO1* was the most significant regulator of osteogenesis in both human and mouse osteoblast. The inhibitory effect of Robo1 knockdown on osteogenic differentiation may be related to the activation of inflammatory signaling pathways.

**Conclusion:**

Our study provides several novel molecular mechanisms involved in the pathogenesis of osteoporosis. *ROBO1* is a potential target for osteoporosis intervention.

## Introduction

Osteoporosis, prevalent among the elderly, is a chronic decondition characterized by reduced bone mass, increased fragility, and heightened susceptibility to fractures (Watts and Manson, 2017). This condition poses a significant threat to the health and longevity of the elderly population. Osteoblasts and osteoclasts are essential cellular components in the regulation of bone metabolism. When osteoclasts-mediated bone resorption exceeds osteoblast-mediated bone formation, the balance of bone remodeling is disturbed, ultimately leading to osteoporosis.

Advancements in omics technologies, including transcriptomics and proteomics, offer opportunities to discover novel molecular targets for osteoporosis. Jemtland et al. highlighted *DKK1*, *SOST*, *SOX4*, *MMP13*, and *MEPE* as differentially expressed genes based on RNA transcriptome studies of iliac bone samples from postmenopausal women (Jemtland et al., 2011). Notably, romosozumab, which targets the *SOST* gene, is now clinically utilized for osteoporosis treatment. However, the heterogeneity of cell types within bone tissue, including osteoblasts, osteocytes, osteoclasts, vascular endothelial cells, neurons, and immune cells (Kular et al., 2012), presents challenges in omics data analysis for identifying genes that determine osteoporosis phenotype. This heterogeneity may lead to uncertainties regarding the cell origin of differentially expressed genes and can result in false negatives due to inconsistent expression changes of a single gene across various cells types, potentially overlooking critical genes. Additionally, many studies have identified key molecules associated with osteoporosis pathogenesis by isolating bone marrow mesenchymal stem cells (BMSC), osteoblasts, and peripheral blood mononuclear cells (PBMC) for transcriptome or proteome analysis (Panach et al., 2020; Roforth et al., 2015; Choi et al., 2017; Song et al., 2017; Zhang L. et al., 2020; Zhang et al., 2016; Qiu et al., 2020; Jin et al., 2018; Zeng et al., 2016; Li et al., 2024). These omics approaches serve as valuable resources for identifying new targets for the regulation and intervention of osteoporosis.

While omics data provides a wealth information on of differentially expressed genes, confirming the role of each gene target in bone mass regulation often requires both *in vivo* and *in vitro* gene knockout studies. The IMPC plays a crucial role in this endeavor, undertaking phenotypic analyses of 20,000 mouse mutants and annotating their functions. This database offers vital support for investigating gene targets associated with human diseases (Groza et al., 2022). Leveraging the IMPC database alongside human disease-related gene expression data, a recent study successfully identified gene targets associated with osteoarthritis, potentially offering new avenues for osteoarthritis treatment (Butterfield et al., 2021). Moreover, Zhang et al. utilized the IMPC database to identify and validate genes involved in the body’s response to external environmental stimuli and biological clock regulation (Zhang T. et al., 2020). Based on the above application of IPMC in screening genetic factors that regulate human diseases and physiological activities, we would like to utilize this database to conduct a preliminary screening of genes associated with bone phenotypes identified in samples from osteoporosis patients.

This study integrates various osteoporosis-related omics datasets, juxtaposes differentially expressed genes/proteins across diverse cell types with genes exhibiting bone metabolism-related phenotypes in the IMPC database, and identified 9 genes including *PPP2R2A*, *RRBP1*, *HSPB6*, *SLC22A15*, *ADAMTS4*, *ATP8B1*, *CTNNB1*, *ROBO1*, and *EFR3B*, which may contribute to pathogenesis of osteoporosis. Through *in vitro* osteogenic differentiation, we further identified *ROBO1* as a conserved regulator of osteogenesis in both human and mouse osteoblast cell lines. The inhibitory effect of osteogenesis may be related to the activation of inflammation in osteoblasts with downregulated of *Robo1*.

## Results

### Overview of osteoporosis-related omics research

We conducted a PubMed search for omics articles focusing on clinical samples of postmenopausal osteoporosis over the past 15 years. Articles with comprehensive data information were selected, and lists of differentially expressed genes or proteins were downloaded. The data were categorized according to the type of clinical sample. Currently, clinical samples are primarily divided into four categories: human bone tissue samples, human osteoblast samples, human BMSC samples, and human PBMC samples, among which osteoblasts and BMSCs are pertinent to osteogenesis. Whole bone tissue samples contain a variety of cell types (Table 1).

**TABLE 1:** Overview of osteoporosis-related omics data.

Publications	Sample	Number of DGE/DEP	Cutoff
Jemtland et al. (2011)	Transiliac bone RNA	497	NA
Li et al. (2024)	Femur protein	630	*P* < 0.05, FC > 1.2
Panach et al. (2020)	Hip osteoblasts RNA	1332	*P* < 0.05, FC > 1.2
Roforth et al. (2015)	hBMSC RNA	279	*P* < 0.05, q < 0.10
Choi et al. (2017)	hBMSC RNA	53	*P* < 0.05, FC > 2
Qiu et al. (2020)	hPBMC RNA	74	NA
Zeng et al. (2016)	hPBMC protein	28	*P* < 0.05, FC > 1.2

NA, not available; FC, fold change.

### Enrichment analysis of bone phenotype-related gene pathways in the IMPC database

**FIGURE 1:**
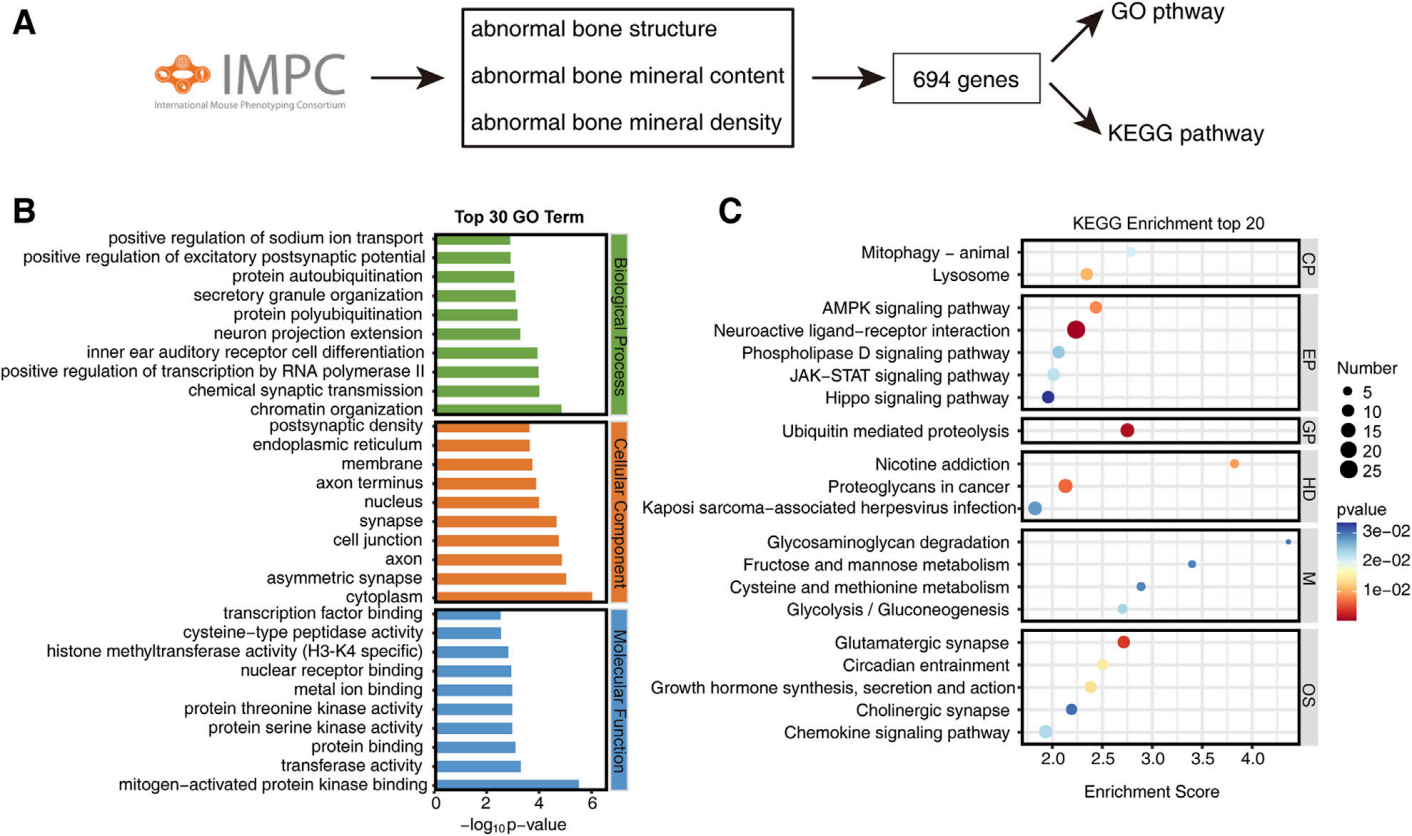
Enrichment of bone phenotype-related gene pathways in the IMPC database. **(A)** Roadmap for screening bone phenotype-related genes from IMPC. **(B)** Top 30 GO enriched pathways of bone phenotype-related genes. **(C)** Top 20 KEGG enriched pathways of bone phenotype-related genes. CP, Cellular processes; EP, Environmental information processing; GP, Genetic information processing; HD, Human diseases; M, Metabolism; OS, Organismal systems.

By querying abnormal bone mineral density, abnormal bone mineral content, and abnormal bone structure in the IMPC database, we obtained a list of 694 genes, along with their corresponding phenotypes and statistical *P*-values (Figure 1A). To elucidate the pathways associated with bone phenotypes, we conducted Gene Ontology (GO) and Kyoto Encyclopedia of Genes and Genomes (KEGG) pathway enrichment analyses on these 694 genes. GO pathway enrichment analysis revealed several enriched pathways across various biological aspects. In terms of biological processes, pathways such as regulation of sodium ion transport, excitatory postsynaptic potential, protein ubiquitination, neural projection, forward transcriptional regulation of RNA polymerase II, chemical synaptic transduction, and chromatin structure were enriched. Regarding cellular components, pathways such as endoplasmic reticulum, cell membrane, axon, nucleus, synapse, cell junction, and cytoplasm showed enrichment. Molecular functions enrichment included MAPK binding, transferase activity, protein binding, protein serine/threonine kinase binding, metal ion binding, nuclear receptor binding, histone methyltransferase (H3-K4), cysteine peptidase activity, and transcription factor binding (Figure 1B). KEGG pathway analysis reveals enrichment of mitophagy and lysosomes in Cellular Processes, while the AMPK signaling pathway, neuro-activating ligand receptor interaction, phospholipase D signaling pathway, JAK-STAT signaling pathway, and Hippo signaling are enriched in Environmental Information Processing. Genetic Information Processing shows enrichment in ubiquitination-mediated protein degradation. Human Diseases exhibit enrichment in nicotine addiction, cancer proteoglycans, and Kaposi’s sarcoma-related viral infection. Metabolism enriched pathways include glycosaminoglycan degradation, fluctuating sugar and mannose metabolism, cysteine and methionine metabolism, and glycolysis/gluconeogenesis. Organismal Systems are enriched in Glutamatergic synapses, rhythm traction, auxin synthesis and secretion, cholinergic pathways and chemokine pathways (Figure 1C).

These findings suggest that bone metabolism diseases may involve a multitude of factors, including neural influences, transcriptional regulation, epigenetic modifications, ubiquitination, biological rhythms, autophagy, hormones, and more.

### Combining omics data with IMPC data to screen target genes

**FIGURE 2:**
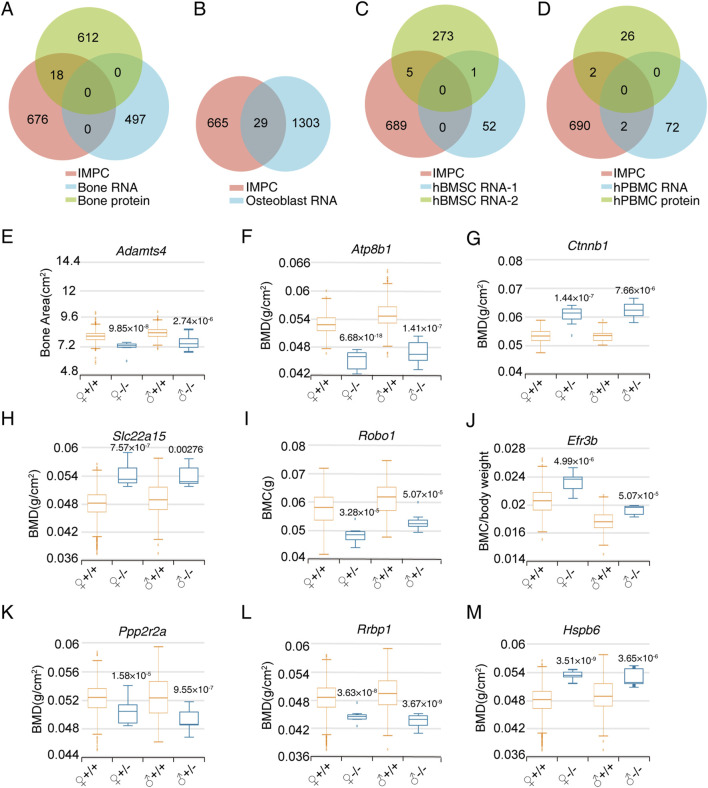
Screening of bone phenotype-related genes in specific cell types based on omics data. **(A)** Venn diagram of IMPC bone phenotype-related genes and differentially expressed genes in bone tissue proteome and RNA transcriptome. **(B)** Venn diagram of differentially expressed genes between IMPC bone phenotype-related genes and osteoblast RNA transcriptome. **(C)** Venn diagram of differentially expressed genes between IMPC bone phenotype-related genes and BMSC RNA transcriptome. **(D)** Venn diagram of differentially expressed genes between IMPC bone phenotype-related genes and PBMC proteome and RNA transcriptome. **(E–M)** Bone phenotype statistical data of the final candidate genes through limited *P* value screening. The sample numbers for every group were as follows. *ADAMTS4*: 633, 7, 595, 8; *ATP8B1*: 352, 8, 369, 8; *CTNNB1*: 30, 10, 38, 12; *SLC22A15*: 2,241, 7, 2,192, 7; *ROBO1*: 324, 8, 321, 8; *EFR3B*: 470, 8, 488, 8; *PPP2R2A*: 418, 7, 410, 7; *RRBP*: 2,633, 7, 2,574, 8; *HSPB6*: 2,241, 8, 2,192, 8. Values represent the mean ± SD, the *P*-values are represented in the figure.

We categorized omics data into four distinct categories: bone tissue, osteoblasts, BMSC and PBMC. By intersecting with the IMPC gene list, we identified 18 differentially expressed genes in bone tissues, 29 in osteoblasts, 6 in BMSC, and 4 in PBMC (Figures 2A–D). Additionally, we identified 9 candidate genes (*PPP2R2A*, *RRBP1*, *HSPB6*, *SLC22A15*, *ADAMTS4*, *ATP8B1*, *CTNNB1*, *ROBO1*, and *EFR3B*) by screening IMPC bone phenotypes with a significance threshold of *P* ≤ 1 × 10^-4 in at least one gender. Gene knockout, whether homozygous or heterozygous (with lethality in homozygotes), resulted in significant alterations in bone density, bone mineral content, or bone structure (Figures 2E–M).

### Verification of siRNA knockdown efficiency

**FIGURE 3:**
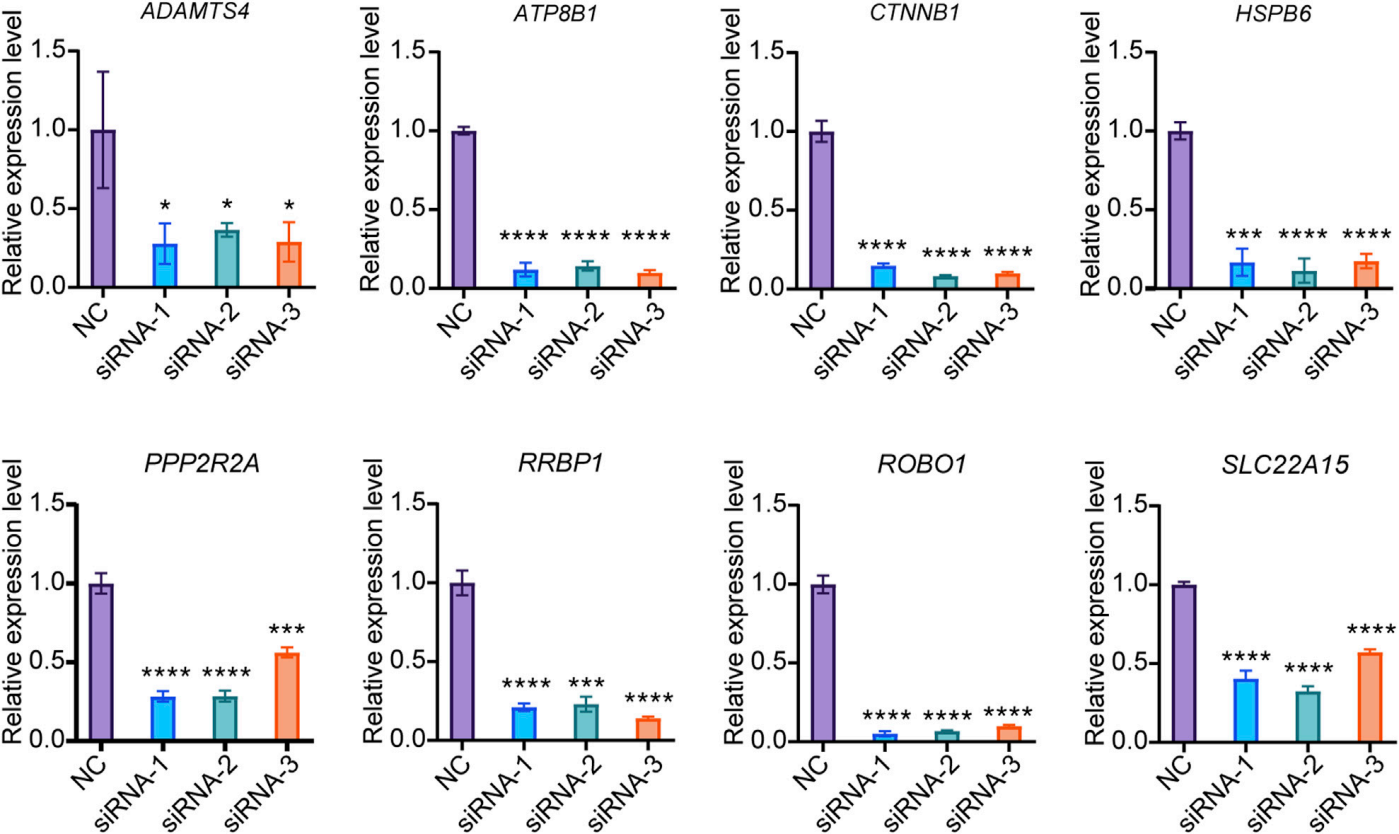
Knockdown efficiency of siRNA. The expression of target genes in Saos-2 cells were detected by qPCR (N = 3). Values represent the mean ± SD. *: *P* < 0.05, ***: *P* < 0.001, ****: *P* < 0.0001.

Since the current drugs for the treatment of osteoporosis mainly target osteoclasts, there are relatively few genetic intervention targets for osteoblasts. This study excluded *EFR3B*, which was screened out from the omics data of PBMCs capable of differentiating into osteoclasts, and focused solely on verifying the functions of the remaining 8 genes in osteogenesis. We developed three small interfering RNAs (siRNAs) targeting each of the 8 candidate genes. In Saos-2 cells, the knockdown efficiency for *PPP2R2A*, *RRBP1*, *HSPB6*, *SLC22A15*, *ADAMTS4*, *ATP8B1*, *CTNNB1*, and *ROBO1* siRNA exceeded 70% (Figure 3). These results indicate the successful screening of siRNA for the candidate genes, paving the way for further research.

### Effect of gene knockdown on osteogenic differentiation of Saos-2

**FIGURE 4:**
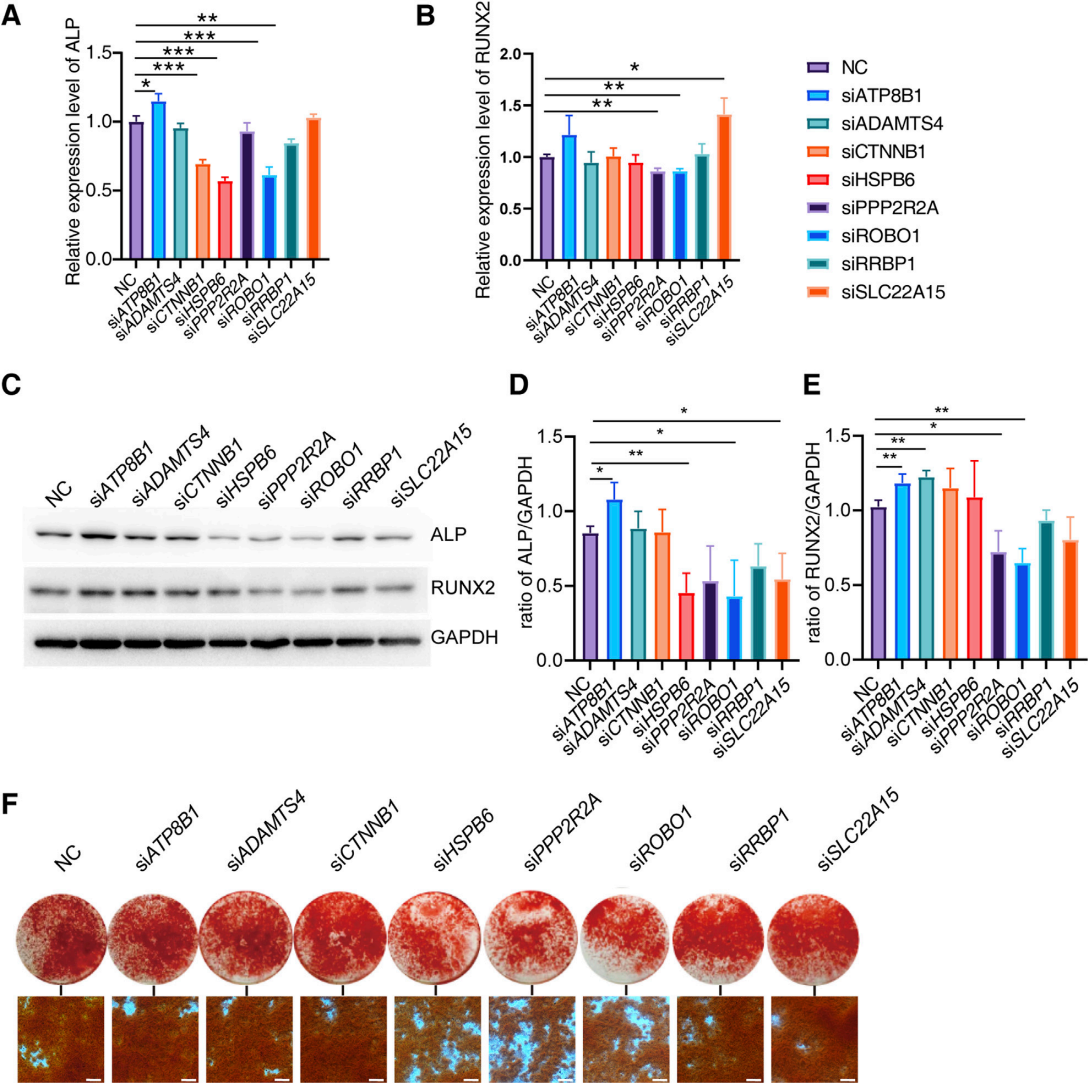
Effect of candidate genes on osteogenic differentiation. **(A, B)** Relative expression of *ALP*, *RUNX2* mRNA in Saos-2 on days 6 of differentiation after candidate gene knockdown (N = 3). **(C–E)** Protein expression and quantification of ALP and RUNX2 in Saos-2 on day 6 of differentiation (N = 3). **(F)** Alizarin red staining of Saos-2 on day 14 of osteogenic differentiation (Scale bar: 200 μm). Values represent the mean ± SD. *: *P* < 0.05, **: *P* < 0.01, ***: *P* < 0.001, ****: *P* < 0.0001.

To investigate the regulatory role of candidate genes in osteogenic differentiation, we induced osteogenic differentiation in Saos-2 cells following siRNA transfection. We evaluated the expression levels of osteogenic differentiation markers, *ALP* and *RUNX2* on the 6th days of differentiation. Our experimental findings reveal that the knockdown of *ATP8B* increases *ALP* expression, whereas the knockdown of *CTNNB*, *HSPB6*, *ROBO1*, and *RRBP1* decreases *ALP* expression. Additionally, the knockdown of *PPP2R2A* and *ROBO1* inhibits the expression of *RUNX2* (Figures 4A, B).

We further examined the expression of ALP and RUNX2 proteins on the 6th day of differentiation. Our results indicate that the knockdown of *ATP8B* significantly increases the expression of both ALP and RUNX2 proteins. Conversely, the knockdown of *PPP2R2A*, *ROBO1*, *RRBP1*, and *SLC22A15* significantly inhibits the expression of ALP and RUNX2 proteins, while HSPB6 alone suppresses the expression of ALP (Figures 4C–E).

To further investigate the impact of candidate genes on mineralization, we knocked down specific genes in Saos-2 cells and conducted osteogenic differentiation. Additionally, we performed Alizarin red staining on the 14th day of differentiation. We found that the knockdown of *HSP60*, *PPP2R2A*, and *ROBO1* genes significantly inhibited Saos-2 mineralization (Figure 4F).

These findings suggest that different genes may have varying effects on osteogenic differentiation at both the transcriptional and translational levels, and their regulation of ALP and RUNX2 may exhibit certain distinctions. Through knockdown experiments, we effectively confirmed the role of specific genes in regulating osteogenic differentiation. Concurrently, we observed discrepancies between the outcomes of gene knockdown and the phenotypic manifestations in knockout mice. This suggests the potential role of these genes in osteoclast functionality as well.

### *Robo1* knockdown inhibits 3T3-E1 osteogenic differentiation

Compared to other candidate genes, the knockdown of *ROBO1* significantly inhibits the osteogenic differentiation at both the RNA and protein levels, and it reduces mineralization in Saos-2 cells. Therefore, we further investigated the role of *Robo1* in mouse osteoblast precursor cells 3T3-E1.

**FIGURE 5:**
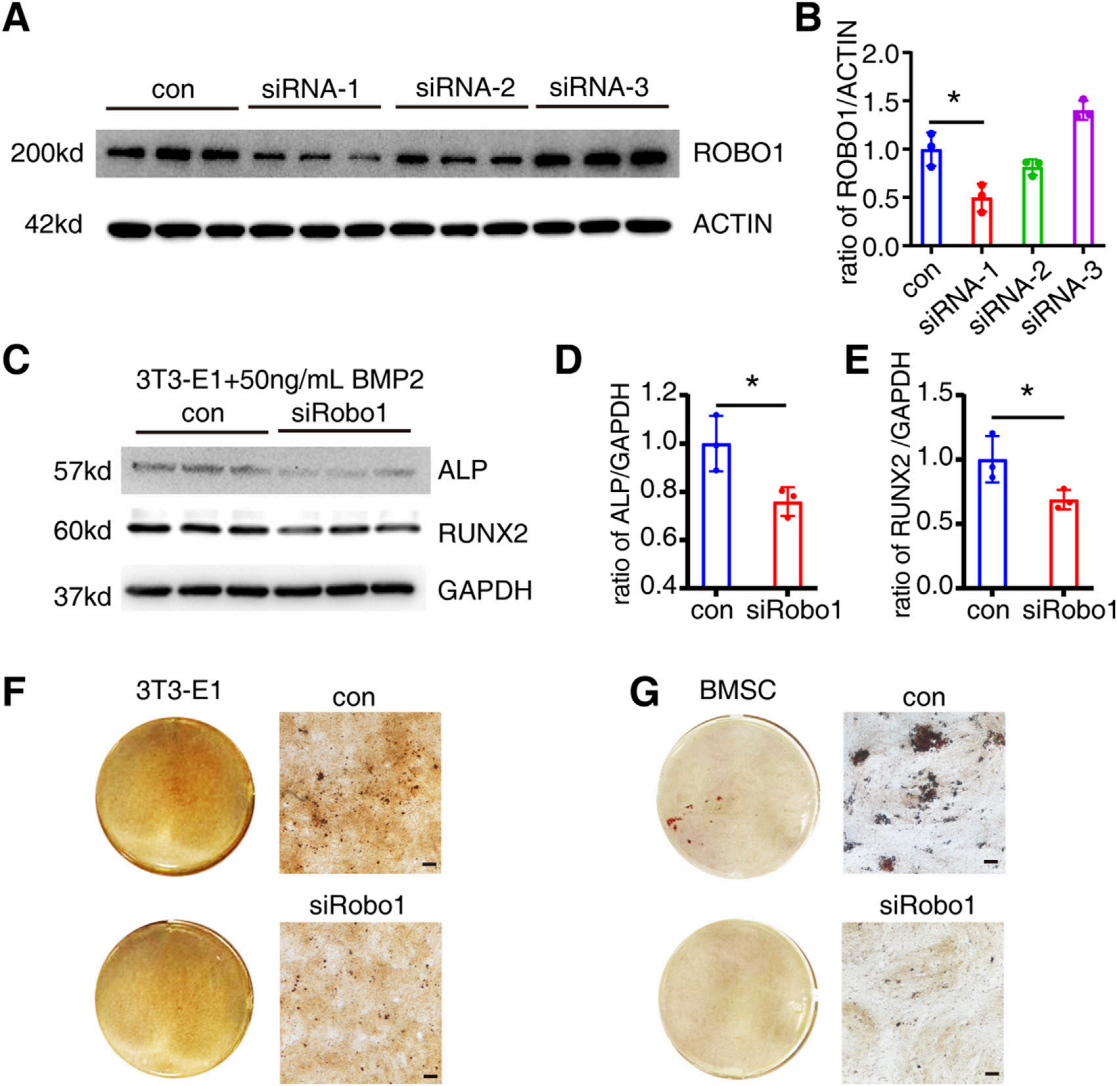
*Robo1* knockdown inhibits osteogenic differentiation of 3T3-E1. **(A, B)** Protein expression and quantification of ROBO1 expression after siRNA transfection 3T3-E1 (N = 3). **(C–E)** Protein expression and quantification of ALP and RUNX2 in 3T3-E1 on day 6 of differentiation (N = 3). **(F)** Alizarin red staining of 3T3-E1. **(G)** Alizarin red staining of BMSC (Scale bar: 100 μm). Values represent the mean ± SD.*: *P* < 0.05.

Similarly, we employed siRNA to knock down the *Robo1* gene in 3T3-E1 cells and confirmed its knockdown effect at the protein level (Figures 5A, B). The siRNA-1 significantly downregulated the ROBO1 expression (*P* = 0.0177). Subsequently, we selected siRNA-1 to transfect 3T3-E1, and detected the expression of ALP and RUNX2 on the 6th day of osteogenic differentiation. The results indicated that *Robo1* knockdown could significantly inhibit the expression of ALP (*P* = 0.0322) and RUNX2 (*P* = 0.0499) (Figures 5C–E). Moreover, we knocked down *Robo1* in 3T3-E1 cells and BMSCs respectively, and performed Alizarin red staining on the 21st day of differentiation. The results showed that the knockdown of *Robo1* significantly inhibits the mineralization function of osteoblasts (Figures 5F, G).

### Regulation of osteogenic differentiation by *Robo1* linked to inflammatory pathways

To further explore the mechanism by which *Robo1* regulates osteogenic differentiation, we knocked down *Robo1* in 3T3 cells, extracted RNA, and performed RNA sequencing (RNA-seq).

**FIGURE 6:**
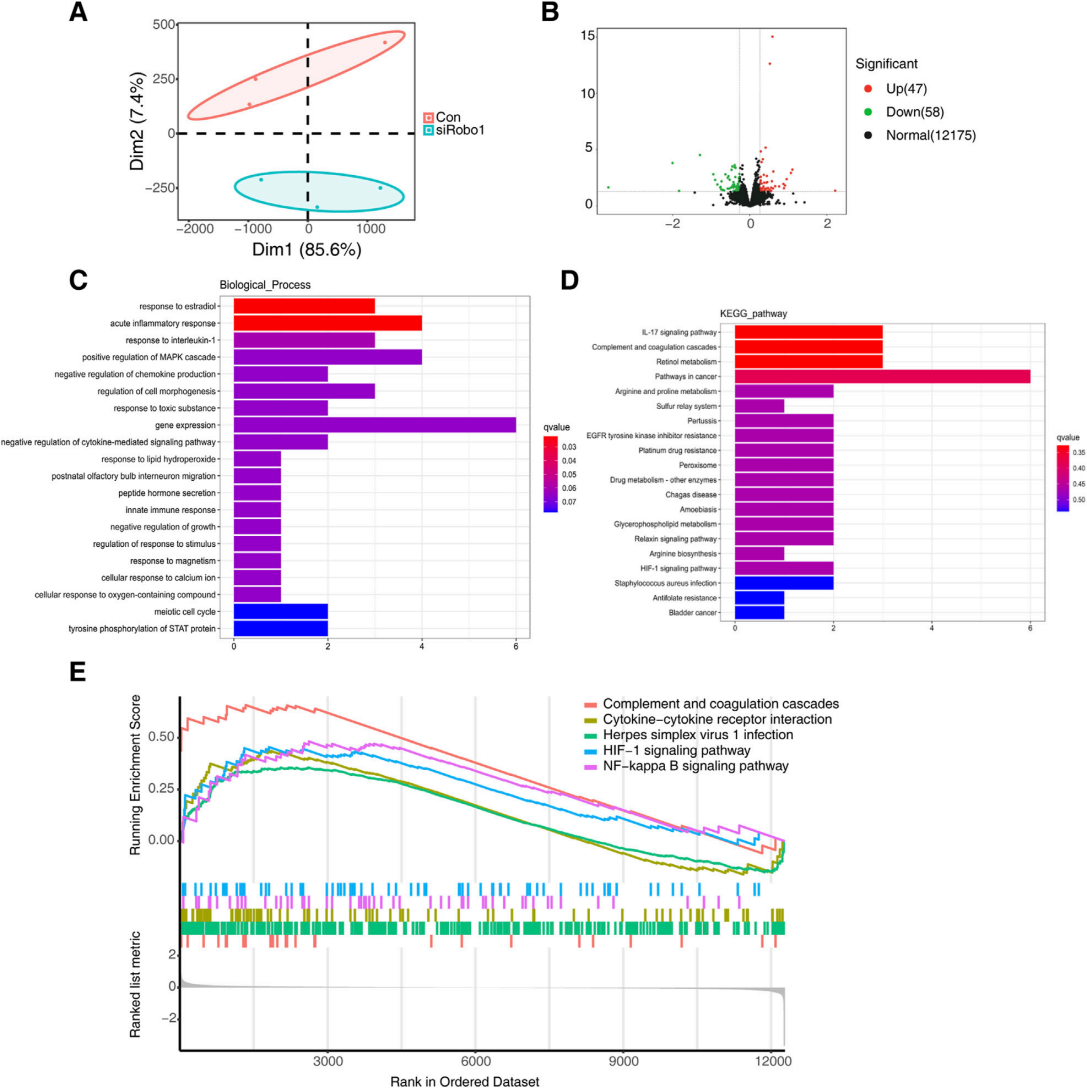
*Robo1* regulates inflammatory pathways in 3T3-E1. **(A)** PCA analysis of control and *Robo1* knocked down samples (N = 3). **(B)** Volcano plot of differentially expressed genes. **(C)** GO enrichment of differentially expressed genes. **(D)** KEGG enrichment of differentially expressed genes. **(E)** GSEA analysis of control and *Robo1* knocked down samples.

The results of the PCA analysis indicated significant differences between the samples in the *Robo1* knockdown group and the control group (Figure 6A). By performing differential expression analysis we identified 47 genes that were upregulated and 58 genes that were downregulated following *Robo1* knockdown (Figure 6B). By performing GO and KEGG pathway enrichment analysis on these 105 differential genes, we found that acute inflammatory response, response to interleukin-1, IL-17 signaling pathway and other inflammation-related pathways were most significantly enriched (Figures 6C, D). Furthermore, the pro-inflammatory factor IL-6 was significantly upregulated after *Robo1* knockdown (Supplementary Table 2). Through GSEA analysis, we found that the top five pathways include complement and coagulation cascades, cytokine-cytokine receptor interaction, herpes simplex virus 1 infection, HlF-1 signaling pathway, and NF-κB signaling pathway, and most of the genes in these pathways were upregulated.

The above results indicate that the knockdown of *Robo1* may activate inflammation-related pathways in osteoblasts, thereby inhibiting osteogenic differentiation and mineralization.

## Discussion

### Clinical translation of osteoporosis intervention based on gene targets

At present, Denosumab, an FDA-approved monoclonal antibody targeting RANKL, has demonstrated effectiveness in long-term clinical treatment of osteoporosis without increasing the risk of cancer, infection, cardiovascular diseases, delayed fracture healing, or hypocalcemia (Bone et al., 2017). Notably, denosumab has been associated with certain side effects in specific populations undergoing anti-osteoporosis treatment, such as inducing hypocalcemia syndrome in patients with cancers and chronic kidney disease (Gouldthorpe et al., 2020; Gopaul et al., 2021), increasing infection risks in patients with chronic kidney disease (Al Adhoubi and Al Salmi, 2021), and elevating the risk of spontaneous vertebral fractures after drug withdrawal (Lamy et al., 2019).

Osteocytes, derived from osteoblasts, constitute the predominant cell type within bone tissue. They play a pivotal role in regulating bone metabolism through the secretion of cytokines and their ability to sense external mechanical stresses (Schaffler et al., 2014; Bonewald and Johnson, 2008). Among the cytokines secreted by osteocytes are DKK1, sclerostin (SOST), RANKL and OPG. Research indicates that knockout of *Dkk1* and *Sost* can lead to increased bone mass (Yee et al., 2018; Witcher et al., 2018; Colditz et al., 2018), highlighting the potential therapeutic significance of targeting these genes in osteoporosis treatment. Omosozumab, a monoclonal antibody against SOST, has received approval and is now utilized in clinical management of osteoporosis. Studies have demonstrated that Romosozumab can promote bone formation, suppress bone resorption, enhance trabecular bone microstructure, and reduce the risk of clinical fractures (Chavassieux et al., 2019; Cosman et al., 2016).

### The potential translational value of new osteoporosis regulatory targets screened out in this study

In this study, omics data served as a valuable resource for identifying candidate genes, while the IPMC database facilitated the verification of gene functions. Through *in vitro* experiments focusing on osteoblast differentiation, we successfully identified *PPP2R2A*, *RRBP1*, *HSPB6*, *SLC22A15*, *ADAMTS4*, *ATP8B1*, *CTNNB1*, and *ROBO1* as potential candidates. These genes exhibit differential expression in patients with osteoporosis. Additionally, the data from gene knockout mouse models in the IMPC database reveal significant alterations in bone phenotypes.

Since the omics data for candidate genes screening was derived from human samples, we hope to first verify the functions of these genes in human osteoblast cell lines, among which Saos-2 is one of the commonly used human cells for studying osteoblast differentiation with more efficiency in forming mineralized nodules than BMSCs or 3T3-E1. The knockdown of *ATP8B1* was found to enhance osteogenic differentiation. Notably, *ATP8B1* knockout mice exhibited a significant decrease in bone mineral density (BMD), possibly indicating a greater influence of osteoclast-mediated bone resorption relative to osteoblast-mediated bone formation. Mechanistically, the regulation of osteoclast differentiation by *ATP8B1* may involve the inflammatory response and macrophage efferocytosis (Yang et al., 2022). In addition, knockdown of *PPP2R2A* and *ROBO1* can significantly inhibit osteogenic differentiation, which is consistent with the phenotype of decreased bone density in gene knockout mice, indicating that *PPP2R2A* and *ROBO1* can affect bone mass changes by regulating osteogenic differentiation. BMD data revealed that *ROBO1* knockout heterozygous mice exhibited a higher degree of bone loss compared to *PPP2R2A* knockout heterozygous mice. Consequently, *ROBO1* emerge as a promising candidate for translational research in osteoporosis intervention based on osteoblast-mediated bone formation.

The bone homeostasis is regulated in the bone microenvironment within the basic multicellular unit (BMU), which contain osteoclasts and osteoblasts together with osteocytes, bone lining cells, osteal macrophages, and vascular endothelial cells (Kular et al., 2012). Several studies have shown that *Robo1* may be involved in the regulation of various cells. In bone tissue, *Robo1* is expressed in vascular endothelial cells, and knocking down *Robo1* in bone marrow endothelial cells can inhibit the promotion of SLIT3 on their cell migration and tube formation (Xu et al., 2018). SLIT2-mediated pro-angiogenesis is also dependent on *Robo1* (Li et al., 2015; Liu et al., 2018). In macrophages, SLIT2/ROBO1 signaling can regulate the cytoskeleton and prevent macropinocytosis and NF-κB activation (Bhosle et al., 2020), suggesting that downregulation of *Robo1* in macrophages can increase inflammatory responses. In excitatory neurons, expression of *Robo1* decrease with aging, indicating that *Robo1* may be involved in the regulation of neuron senescence (Zhang et al., 2022). Although *Robo1* is rarely expressed in osteoclasts (Xu et al., 2018), bone marrow-derived macrophages (BMMs) *Robo1* knockdown inhibits SLIT2-mediated osteoclast differentiation, suggesting that *Robo1* may be directly involved in the regulation of osteoclast differentiation (Park et al., 2019). *In vitro* studies have found that *Robo1* knockdown can promote osteoclast differentiation, but has no effect on osteoblast migration (Kim et al., 2018). Thus, the role of *Robo1* in regulating osteogenic differentiation and osteoblast function remains unclear. It has been reported that *Robo2* can regulate the osteogenic differentiation of 3T3-E1 by modulating autophagy (Yuan et al., 2023), suggesting that *Robo1* may also be involved in the regulation of osteogenic differentiation through a similar pathway.

*In vivo* studies have demonstrated that *Robo1* ^−/−^ mice exhibit a significantly reduced trabecular bone mass, trabecular thickness, and trabecular number and a higher trabecular separation than their wild-type (WT) littermates. The bone formation rate was lower, and the bone resorption parameters were higher in *Robo1* ^−/−^ mice than in WT (Xu et al., 2018; Kim et al., 2018). Although *Robo1* may affect bone homeostasis in various ways, the construction of osteoblast-specific *Robo1* knockout mice can provide *in vivo* evidence that *Robo1* affects bone mass by regulating osteogenesis mediated by osteoblasts.

### *ROBO1* and inflammatory response

This study used RNA-seq to preliminarily explore the potential mechanism of *Robo1* regulating osteoblasts. The results suggested that inflammatory-related signaling pathways were activated after *Robo1* knockdown. We found that the proinflammatory factor IL6 was significantly upregulated after *Robo1* knockdown, and many studies have shown that IL6 can inhibit osteogenic differentiation (Kaneshiro et al., 2014; Peruzzi et al., 2012), indicating that the weakening of osteogenic differentiation caused by *Robo1* knockdown may be related to the increased expression of IL6. Previous studies have shown that reactive oxygen species (ROS) can inhibit osteoblast differentiation and activity by inducing inflammatory response (Marques-Carvalho et al., 2023; Iantomasi et al., 2023). Our results showed that the peroxisome pathway was enriched in the KEGG analysis, and the HIF-1 signaling pathway was enriched in the GSEA analysis, suggesting that the activation of redox-related signaling pathways may be related to the inflammatory response of osteoblasts.

### Summary

Through the results of omics sequencing of clinical samples and screening of IMPC, this study successfully screened *ROBO1* as a key molecule regulating osteoporosis, and *ROBO1* downregulation may promotes osteoporosis by inhibiting osteoblast differentiation and mineralization. Our study provides several novel molecular targets involved in the pathogenesis of osteoporosis. Further research on these molecular targets is expected to provide a new approach for the clinical intervention and treatment of osteoporosis, such as developing agonists, inhibitors or antibodies against gene targets.

## Materials and methods

### Cell culture

Saos-2, 3T3-E1, BMSC were obtained from Procell Biological Company and cultured in a 37°C incubator containing 5% CO2. Saos-2 was cultured using McCoy’s 5A medium containing 15% FBS and 1% P/S. 3T3-E1 and BMSC were cultured using αMEM medium containing 10% FBS and 1% P/S.

### siRNA transfection and qPCR

Use DEPC water to dissolve siRNA (Shenggong) to a final concentration of 10 nM. Use Lipofectamine RNAiMAX for RNA transfection according to the instructions. Change the medium within 24 h of transfection. After continuing to culture for 48 h, use an RNA extraction kit (Yeason) to extract cellular RNA.

The RNA concentration was determined by a NanoDrop (Thermo), and the cDNA was synthesized using a NovoScript Plus All-in-one 1st Strand cDNA Synthesis SuperMix (Novoprotein). Quantitative real-time PCR was performed using NovoStart SYBR qPCR SuperMix plus (Novoprotein) with StepOne Plus (Applied Biosystems). The relative levels of target genes were normalized to *Gapdh*. Primer information for Q-PCR is included in the Supplementary Table 1.

### Osteogenic differentiation

Add 10 mM β-Glycerophosphate disodium salt hydrate (Abmole), 50 ug/ml VC (Abmole) and 10 nM dexamethasone (MCE) to the growth medium to prepare an osteogenic differentiation medium. When the cells grew to 80% confluence, siRNA transfection was performed. The differentiation medium was replaced the next day and the medium was changed every 2 days.

### Western blot

The cells were washed with PBS for once before adding RIPA. The cells were lysed on ice for 30 min and the lysate was collected. After centrifuging at 4°C and 13,000 rpm for 10 min, supernatant was collected. BCA method was used to determine the protein concentration. After SDS-PAGE electrophoresis, the protein was transferred to a PVDF membrane, then blocked with 5% milk. Membranes were incubated with primary antibodies (ALP: Affinity DF6225 1:1,000, RUNX2: proteintech 20700-1-AP 1:1,000, GAPDH: proteintech 60004-1-Ig 1:3,000, ACTIN: proteintech 23660-1-AP 1:3,000) overnight at 4°C. After washed three times with TBST, membranes were incubated with secondary antibodies (proteintech SA00001-1 1:10,000, proteintech SA00001-2 1:10,000) at room temperature for 1 h. Membranes were then visualized with chemiluminescence imager (Tannon). Use ImageJ software for protein quantification.

### Alizarin red staining

On the 14th day of Saos-2 differentiation, and the 20th day of 3T3-E1 and BMSC differentiation, the osteogenic mineralization level was detected using the Alizarin red staining kit. Wash the cells once with PBS, fix the cells with 4% PFA at room temperature for 20 min, and then wash the cells three times with PBS. Add an appropriate amount of Alizarin red S staining solution to evenly cover the cells, and stain at room temperature for 30 min. Wash thoroughly with distilled water, and then take pictures under a microscope.

### RNA seq and enrichment analysis

RNA-seq was performed on an MGI platform at the Tsingke Biotechnology Co., Ltd. Differential expression analysis of two groups was performed using the DESeq2 R package (1.26.0). The screening criteria for this project were: Fold Change ≥ 1.2 and *P* value < 0.05. We used KOBAS (Mao et al., 2005) software to test the statistical enrichment of differential expression genes in KEGG pathways. Gene Ontology (GO) enrichment analysis of the differentially expressed genes (DEGs) was implemented by the GOseq R packages based Wallenius non-central hyper-geometric distribution (Young et al., 2010), which can adjust for gene length bias in DEGs. The GSEA analysis in this project uses the gene sets of KEGG pathways and GO BP, CC, and MF branches as the gene sets of interest, and uses the log2FC of each differential group as the score of the background gene set to analyze the enrichment of the gene set of interest.

### Statistical analysis

The statistical analyses were performed using Student’s t-tests using GraphPad Prism 8. *P* < 0.05 was considered statistically significant (*P < 0.05, **P < 0.01, and ***P < 0.001).

## Data Availability

The data presented in this study are deposited in the NCBI BioProject repository, accession number PRJNA1124862.
