# Supplementary material for: IMPC-based screening revealed that ROBO1 can regulate osteoporosis by inhibiting osteogenic differentiation
Source: Front Cell Dev Biol. 2024 Oct 8;12:1450215. doi: 10.3389/fcell.2024.1450215 (PMC11494888; doi:10.3389/fcell.2024.1450215)
Supplement: Supplementary file 2 [file Table1.DOCX]

| Genes | Forword | Reverse |
| --- | --- | --- |
| *ADAMTS4* | TCACTGACTTCCTGGACAATGGC | GGTCAGCATCATAGTCCTTGCC |
| *ATP8B1* | CTTCTTGCTCGCAGTTTGCCAC | GCCAAAGTTCCTGGCAGCGTTT |
| *CTNNB1* | CACAAGCAGAGTGCTGAAGGTG | GATTCCTGAGAGTCCAAAGACAG |
| *HSPB6* | GCCACTTTTCGGTGCTGCTAGA | GCGCGACGAATCCGTGCTCAT |
| *PPP2R2A* | GCAACAGGAGATAAAGGTGGTAG | TGGTTCATGGCTCTGGAAGGTG |
| *RRBP1* | TCCTGTCTGAGAAGGCTGGCAT | CCTCAGTTTGCTCTTGGCGACA |
| *ROBO1* | AGTGAGCCTCAGTTCATCCAGC | GCTCCAATACCTGCTATGAAGGC |
| *SLC22A15* | CAGGGAACGGTGGTCTTTCTCT | TGAGTTTGCGGTTCCTCTTGGC |
| *GAPDH* | GTCTCCTCTGACTTCAACAGCG | ACCACCCTGTTGCTGTAGCCAA |
